# Works Published by the Sydenham Society

**Published:** 1856-11

**Authors:** 


☞ As it may interest some of our readers to know where copies of the works published by the Sydenham Society may be obtained, we copy the following from a circular sent us:—

The Sydenham Society, of London.—List of the Society's Works, of which copies are still on hand, and from which new members, subscribing for the current year, may make a selection, on payment of an additional five dollars for any three vols., with the exception of those to which an asterisk is affixed. Those to which an asterisk is affixed, or any other single volume, may be had for $2 50 per volume.

Sydenhami Opera Omnia. 1 vol.

Hasse's Pathological Anatomy. 1 vol.

Rhazes on the Small-pox and Measles. 1 vol.
The Works of Hewson. Portrait and plates. 1 vol.
Dupuytren's Lectures on Diseases and Injuries of Bones. 1 vol.
Dupuytren on Lesions of the Vascular System, &c. 1 vol.
Memoirs of the French Academy of Surgery. 1 vol.
Feuchtersleben's Medical Psychology. 1 vol.
Microscopical Researches of Schwann and Schleiden. 1 vol. Plates.
The Works of W. Harvey, M.D. 1 vol.
The Genuine Works of Hippocrates. 2 vols.
Essays on Puerperal Fever, and other Diseases peculiar to Women. 1 vol.
The Works of Sydenham, translated from the Latin. 2 vols.
Unzer and Prochaska on the Nervous System. 1 vol.
Annals of Influenza. 1 vol.
Romberg on Diseases of the Nervous System. 2 vols.
Kolliker's Manuel of Human Histology. 2 vols. Wood-cuts.
*Rokitansky's Pathological Anatomy. Complete in 4 vols.
*Hunter on the Gravid Uterus. 1 vol. folio. 34 plates with descriptive letter-press.
Wedl's Pathological Histology. 1 vol. Wood-cuts.
Oesterlen's Medical Logic. 1 vol.
Velpeau on Diseases of the Breast. 1 vol.
The Works of Aretæus, Greek and English. 1 vol.

The subscription, constituting a member, is five dollars annually, payable in advance.

Three volumes, handsomely bound in a uniform manner in cloth, gilt edged, are usually issued in the year.

RICHARD J. DUNGLINSON, M.D.,
*Hon. Local Secretary for Philadelphia.*

---

*The Use of Glycerine for the Preservation of Organic Bodies.*

Luton states, that animal and vegetable substances may be kept for a long period perfectly free from decomposition when immersed in glycerine. He also finds that it is a good antiseptic agent for injecting dead bodies.—*London Lancet.*

---

*Poisoning by Chloroform.*

The most extraordinary over-dose of chloroform yet known, was wilfully swallowed by a patient recently in London. The man drank about four ounces at one draught! Wild intoxication, followed by profound insensibility, ensued; but, after various relapses and accidents, he is now quite well.—*London Lancet.*

*Transactions of the Illinois State Medical Society.*

We are glad to see by our exchanges that the past year's doings of our State Society are regarded abroad as highly creditable; and, as it may encourage many of the members to make renewed efforts and more extended investigations, we copy the following from that well-known sheet the *Boston Medical and Surgical Journal*:—

'*Transactions of the Illinois State Medical Society for the Year* 1856. Pp. 92. Chicago, 1856.—No surer test of the rapid advance of the most favored of the more Westerly States, in population and influence, could be afforded, than is furnished by a glance at the condition of medical science in that State, as shown by the transactions of its Medical Society, which has just met for the sixth time since its formation. This Society, it appears, numbers about fifty active members, who assemble each year, in one of the principal towns of the State, for the purpose of medical improvement and social intercourse. The pamphlet before us contains, in addition to the business doings of the Society, an excellent report from the Committee on Practical Medicine, by Dr. Thompson; a report on Orthopœdic Surgery; and the Annual Address by the President of the Society, Dr. Davis.

'The report of Dr. Thompson is clear and able, containing much useful information in reference to the diseases which have been most prevalent during the past year. Diseases of a periodic nature, as we should expect, come first in order. This class of diseases, it seems, "have been more extensively prevalent during 1855, than at any previous period;" in Clark County showing itself on the *barrens*, which have before been exempt from its ravages. In the treatment of these diseases, Dr. Thompson mentions the sulphate of cinchona as possessing "many if not equal advantages with the quinine," not producing the unpleasant cerebral sensations peculiar to the latter remedy, and being cheaper and more easily obtained.

'Appended to the remarks on Periodic Fevers, is a carefully-prepared table, containing 356 cases of periodic disease, by which it appears that the notion entertained by some systematic writers, that the paroxysm of quotidian is early in the morning, is not infallible. They also show that these fevers do not always assume a graver form as the season advances, as has by many been supposed; the severest cases having occurred in September and October, while in the later months "the diseases were simple intermittents, and soon dismissed."

'Dr. T. prefers large doses of quinine (from six to nine grains combined with morphine,) stating that it produces less excitement than when given in smaller doses at short intervals.

'The other diseases alluded to in the report are typhoid fever, pneumonia, the exanthemata (more particularly variola, which of late seems to have prevailed to an unusual extent there, as elsewhere,) milk-sickness, and cholera.

'We pass over the able report on Orthopœdic Surgery, by Dr. Prince, to the Annual Address by the President of the Society, Dr. Davis, which is mainly taken up with the treatment of phthisis by alcoholic liquids. Dr. Davis is inclined to doubt the opinion which has of late obtained, that whisky punch is a specific in this formidable disease, and adduces much evidence and brings much good sense to bear upon this important question. Whatever be the present apparent success of this mode of treatment, it certainly must require a large experience to test the merits of a method which seems at variance with the deductions of the most careful and varied experimental physiological researches.

'Dr. D. concludes by entreating the members of the Society to "adopt more rigorous and careful modes of investigation; at least to avoid giving currency to those loose general statements which can only deceive the sick, and sometimes give encouragement to habits which may plunge thousands of the well into irretrievable ruin."

'We cannot take leave of this subject without the expression of a hope that some of the State Societies nearer home will copy the example of our brethren of Illinois, and render their annual meeting one of profit as well as of festivity. This noble State, extending as it does from the parallel of Boston to that of Norfolk, affords a rich field for the observing and well-educated physician, and with its present corps of medical officers must ultimately yield fruits which may be of inestimable benefit to the race.' F. E. O.

